# City Sanitation

**Published:** 1914-08

**Authors:** 


CITY SANITATION.—A great educational campaign is being conducted throughout the country to induce cities and towns to clean up and stay clean. The aphorism that cleanliness is akin to godliness is being interpreted to apply to communities as well as to individuals, and to one's surroundings as well as to his person. It is a lesson that will require time in the teaching, because for many generations it has gone unheeded. It is right that in this State credit should be given largely to *Holland's Magazine* for so actively pushing, through contests and frequent inspections, this great health movement, and to Dr. M. M. Carrick, who has proven such an efficient sanitary expert, and who has so ably directed the work undertaken by *Holland's*.

The good results of the movement did not stop when the contests closed, but are still being seen. Quite recently Temple, one of the progressive cities of the State, became interested in municipal cleanliness, and employed Dr. Carrick to show the people just how dirty, how unhealthfully dirty, the city really was, and then to show the people how to clean up and stay clean. Dr. Carrick did not mince matters. He told the Temple folks in plain language that he had to hold his nose on his tours of inspection, and did not have to get far from the very center of the city before grabbing his nose. With good eyes, and even poor nostrils, Temple's dirt could be seen and smelled.

The people were interested, and the officials at once said: "Show us what to do, and we will do it." The result was that in a few days alleys that had been reeking with filth became models of cleanliness, and six wagons were kept busy hauling away the filth that was pointed out, until all the alleys were as clean as the down-town streets. Then private yards were inspected, and the owners were told to clean up; tin cans were removed from vacant lots, weeds and rubbish were gathered and burned; hotels, restaurants, meat markets, dairies, supply stores and all places handling provisions were visited, and the owners were told that cleanliness would in future be considered as akin to business, and that a hint unobserved would be as bad as a boycott. So these places all cleaned up, and now Temple is feeling somewhat like a copy of the original "spotless town." More than that, the city officials are so proud of it that they say they are going to make laws that will keep Temple clean.

What has been done in Temple can be done and should be done in other places. There is no more excuse for a city to be unclean than for an individual to stay unwashed. City officials owe it to the people who are careful of their personal surroundings to require the careless and indifferent to respect their rights. No person has a right to permit conditions on or about his premises that will impair the health of his neighbor or the community. It is hoped that Temple's example may be followed in every part of the country.

